# Environmental drivers and spatial scaling of species abundance distributions in Palaearctic grassland vegetation

**DOI:** 10.1002/ecy.3725

**Published:** 2022-05-28

**Authors:** Werner Ulrich, Thomas J. Matthews, Idoia Biurrun, Juan Antonio Campos, Patryk Czortek, Iwona Dembicz, Franz Essl, Goffredo Filibeck, Gian‐Pietro Giusso del Galdo, Behlül Güler, Alireza Naqinezhad, Péter Török, Jürgen Dengler

**Affiliations:** ^1^ Department of Ecology and Biogeography Nicolaus Copernicus University Toruń Poland; ^2^ GEES (School of Geography, Earth and Environmental Sciences) and Birmingham Institute of Forest Research University of Birmingham Birmingham UK; ^3^ cE3c – Centre for Ecology, Evolution and Environmental Changes/Azorean Biodiversity Group/CHANGE – Global Change and Sustainability Institute and Universidade dos Açores (FCAA) Angra do Heroísmo Portugal; ^4^ Department of Plant Biology and Ecology University of the Basque Country UPV/EHU Bilbao Spain; ^5^ Białowieża Geobotanical Station, Faculty of Biology University of Warsaw Białowieża Poland; ^6^ Department of Ecology and Environmental Conservation, Institute of Environmental Biology, Faculty of Biology University of Warsaw Warsaw Poland; ^7^ Bioinvasions, Global Change, Macroecology Group, Department of Botany and Biodiversity Research University of Vienna Vienna Austria; ^8^ Department of Agriculture and Forest Science (DAFNE) University of Tuscia Viterbo Italy; ^9^ Department of Biological, Geological and Environmental Sciences University of Catania Catania Italy; ^10^ Biology Education Dokuz Eylul University İzmir Turkey; ^11^ Department of Plant Biology, Faculty of Basic Sciences University of Mazandaran Mazandaran Iran; ^12^ MTA‐DE Lendület Functional and Restoration Ecology Research Group University of Debrecen Debrecen Hungary; ^13^ Polish Academy of Sciences, Botanical Garden ‐ Center for Biological Diversity Conservation in Powsin Warszawa Poland; ^14^ Department of Ecology University of Debrecen Debrecen Hungary; ^15^ Vegetation Ecology, Institute of Natural Resource Management (IUNR) Zurich University of Applied Sciences (ZHAW) Wädenswil Switzerland; ^16^ Plant Ecology, Bayreuth Center for Ecology and Environmental Research (BayCEER) University of Bayreuth Bayreuth Germany

**Keywords:** lognormal distribution, log‐series distribution, Palaearctic grassland, plant cover, spatial scaling, species abundance, Weibull distribution

## Abstract

Species abundance distributions (SADs) link species richness with species abundances and are an important tool in the quantitative analysis of ecological communities. Niche‐based and sample‐based SAD models predict different spatial scaling properties of SAD parameters. However, empirical research on SAD scaling properties is largely missing. Here we extracted percentage cover values of all occurring vascular plants as proxies of their abundance in 1725 10‐m^2^ plots from the GrassPlot database, covering 47 regional data sets of 19 different grasslands and other open vegetation types of the Palaearctic biogeographic realm. For each plot, we fitted the Weibull distribution, a model that is able to effectively mimic other distributions like the log‐series and lognormal, to the species–log abundance rank order distribution. We calculated the skewness and kurtosis of the empirical distributions and linked these moments, along with the shape and scale parameters of the Weibull distribution, to plot climatic and soil characteristics. The Weibull distribution provided excellent fits to grassland plant communities and identified four basic types of communities characterized by different degrees of dominance. Shape and scale parameter values of local communities on poorer soils were largely in accordance with log‐series distributions. Proportions of subdominant species tended to be lower than predicted by the standard lognormal SAD. Successive accumulation of plots of the same vegetation type yielded nonlinear spatial scaling of SAD moments and Weibull parameters. This scaling was largely independent of environmental correlates and geographic plot position. Our findings caution against simple generalizations about the mechanisms that generate SADs. We argue that in grasslands, lognormal‐type SADs tend to prevail within a wider range of environmental conditions, including more extreme habitats such as arid environments. In contrast, log‐series distributions are mainly restricted to comparatively species‐rich communities on humid and fertile soils.

## INTRODUCTION

More than 80 years after the seminal work of Motomura (1932), the concept of the species abundance distribution (SAD) in ecological communities remains a focus of ecological interest (Matthews & Whittaker, 2014; McGill et al., 2007; Ulrich et al., 2010). SADs link species richness with relative species abundances and exhibit a consistent general form with many rare and few abundant species (McGill et al., 2007). They are important in the quantitative analysis of ecological communities, particularly in the quantification of rarity (Kunin, 1997), competitive hierarchies (Mac Nally et al., 2014), niche partitioning (Sugihara et al., 2003; Tokeshi, 1999), changes in species functional traits (Dantas de Miranda et al., 2019), and the concept of neutral community assembly (Hubbell, 2001; May, 1975). Recent interest has shifted from statistical distribution fitting (Alonso et al., 2008; Baldridge et al., 2016; Morlon et al., 2009; Ulrich et al., 2010) and the testing of the underlying niche‐based and stochastic theories (Connolly et al., 2005; Magurran & Henderson, 2003) toward the analysis of observed and predicted changes in relative abundances across spatial (Borda‐De‐Água, et al., 2012; Ferreira de Lima et al., 2020; Šizling et al., 2009) and temporal (Tomašových & Kidwell, 2010) scales. A greater understanding of the scaling of SADs and the functional consequences of SAD scaling patterns are not just of theoretical interest, and will likely be useful in biodiversity management (Matthews & Whittaker, 2015).

Competitive and niche‐orientated approaches have often assumed SADs to be generic properties of ecological communities determined by species interactions (Centurión & López Gappa, 2011; Tokeshi, 1999) and niche partitioning (Sugihara, 1980). Niche‐orientated SAD model parameters are determined by species richness and the specific pattern of niche division, but not by the temporal or spatial dynamics of community assembly. However, sample‐based theoretical work has demonstrated that the parameters of important SAD models change with increasing sample size (Green & Plotkin, 2007; Šizling et al., 2009) and patterns of spatial aggregation (Dornelas & Connolly, 2008). These models include the exponential series, characterized by identical proportions of species along the gradient of log‐transformed species abundance (Motomura, 1932), the log‐series sample distribution, characterized by a few highly abundant species and an excess of species represented by a single individual (Fisher et al., 1943), and the lognormal distribution, characterized by a comparatively high number of species with intermediate abundance and similar numbers of relatively abundant and rare species (Gaston & Blackburn, 2000; Preston, 1948). Importantly, Locey and White (2013) demonstrated that the shape of SADs is determined by the interplay of the total numbers of individuals and species. Both increase with increasing sample area. Therefore, the sample behavior of SADs should automatically translate into changes in SAD shape across spatial scales. The situation is complicated by the fact that local communities are not simply random samples from the larger regional species pools. Instead, they result from three basic processes: species‐specific dispersal, habitat filtering, and local species interactions (e.g., D'Amen et al., 2017; Török et al., 2018; Vellend, 2016). These three processes operate differently at different spatial scales. As such, for this reason, we also cannot expect SADs to be invariant of spatial scale.

At spatial extents above the local community, two contrasting theoretical approaches predict different SAD shapes. Neutral approaches generally predict that log‐series SADs will characterize regional species pools (Hubbell, 2001), as reported by Wu et al. (2019). In contrast, Connolly et al. (2005) reported scale‐invariant lognormal regional SADs of exhaustively sampled marine fish and coral reef communities, arguing that the observed invariance results from the corresponding scaling of multiple ecological processes that force SADs into a lognormal shape. We note that Šizling et al. (2009) argued against exact scale invariance of lognormal SADs. These authors showed that, if the SAD at one scale is lognormal, the SADs at other scales converge on right‐skewed distributions that can appear roughly lognormal, resulting in apparent scale invariance.

Across taxa, studies have reported changes in the (i) parameters of the models that best fit local SADs with increasing spatial scale and (ii) type of SAD model that provides the most accurate representation of the empirical distribution (in what follows referred to as the SAD shape). For example, Borda‐de‐Água et al. (2017) reported that the variance and skewness of arthropod SADs changed predictably according to an allometric function along spatial gradients. Wu et al. (2019) found consistent directional temporal changes in initially variably shaped local forest tree SADs toward regional log‐series distributions as predicted by neutral, dispersal‐driven models (Hubbell, 2001). Ferreira de Lima et al. (2020) identified a decreasing hierarchy of factors that trigger variability in Brazilian Atlantic forest SAD shapes across spatial and temporal scales: sample size, conspecific aggregation, and β‐diversity. Antão et al. (2021) found that Poisson lognormal models, including those with multiple modes, provided the best fit to large‐scale SADs of multiple taxa (the log‐series never provided the best fit to the largest‐scale SADs), whereas a mix of log‐series and Poisson lognormal models provided the best fits to the SADs of smaller areas.

This prior work has not resolved the question of whether observed changes in SAD shapes with grain size and in response to the aforementioned three basic processes (interactions, filtering, dispersal) occur in a predictable way and whether they are taxon‐ or community‐specific. It also remains unclear whether SAD scaling involves gradual changes in parameter values within the same type of distribution or, instead, large and abrupt shifts (i.e., breakpoints) in parameter values and thus switching between different types of distributions. Sample theory (Green & Plotkin, 2007) assumes gradual changes in SAD model parameter values within the same general distribution shape, whereas certain neutral models (e.g., Hubbell, 2001) predict larger shifts from regional log‐series SADs toward local lognormal‐type distributions, depending on the degree of dispersal limitation. Such changes in relative abundance have strong implications for the scaling of the ecological processes that determine the hierarchy of species abundances. An empirical assessment of the type of scaling and the respective scaling parameters would allow for an improved extrapolation of observed abundance distributions.

Except for the influence of dispersal, few empirical studies have dealt with the ecological drivers that influence the spatial scaling of SADs, particularly for plants. Global comparisons of woody (Matthews et al., 2019; Ulrich et al., 2018) and dryland plants (Ulrich et al., 2016) and local comparisons of forest gaps (Salvador‐van Eysenrode et al., 2003) have highlighted the importance of climatic variability and environmental stress. Work on other plant groups is lacking, as are scaling analyses focused on fine, local‐scale SAD data. Here we fill this knowledge gap by focusing on extra‐tropical grasslands and other open vegetation communities. We use an exceptionally large Palaearctic data set, the GrassPlot database (Biurrun et al., 2019, 2021; Dengler et al., 2018), to address the questions around the scaling of abundance distributions, using percentage cover estimates as proxies of abundance. The GrassPlot data stem from diverse site conditions (e.g., from sea level to more than 5000 m above sea level [a.s.l.], from very wet to very dry sites, and from humid to semiarid climates) and management regimes (e.g., natural, seminatural, intensified) (Dengler et al., 2020). This variation makes it possible to link the observed changes in SAD parameter values to environmental characteristics. Importantly, our data allow us to study the scaling of SADs within identical vegetation types and to compare the patterns of scaling among vegetation types.

Based on the preceding discussion of empirical and sample theoretical predictions, we examine (i) which types of SADs are realized in extra‐tropical grasslands, (ii) how SAD shape changes across environmental gradients across the Palaearctic, and (iii) whether and how the scaling of SADs along spatial gradients might influence inferences of SAD variability at larger, geographical spatial scales.

## MATERIALS AND METHODS

### Vegetation‐plot data

We compiled data from 3531 plots across 56 data sets from the collaborative vegetation‐plot database GrassPlot (Biurrun et al., 2019; Dengler et al., 2018, https://edgg.org/databases/GrassPlot). Using a minimum species richness threshold of 20 to filter these data, we extracted vascular plant data from 1725 single plots across 47 data sets each covering an area of 10 m^2^ (data sets, metadata, and references in Ulrich et al., 2021). In total, these plots come from 20 different countries in Europe and Asia (Appendix S1: Figure S1) and cover 19 broad vegetation types (2nd level of the ecological‐physiognomic typology of GrassPlot; Biurrun et al., 2019). The lower richness boundary (20) allowed for sufficiently precise SAD fits and enabled us to assess the change in community parameters along gradients of increasing richness and abundance (cf. Ulrich et al., 2010). Abundances for all species in a plot were assessed by the percentage cover (typically used in plant SAD studies rather than actual abundances) (Anderson et al. 2012; Chiarucci et al., 1999). Cover data are often more strongly correlated with plant biomass than with the number of ramets, that is, single shoots (Chiarucci et al., 1999). Therefore, cover‐based SADs are particularly effective at quantifying the distribution of plant species biomass within and across vegetation plots.

For the analysis of spatial SAD scaling, we selected 40 plot clusters from the data sets, that is, groups of plots from the original data set with identical vegetation type that contained at least 15 individual plots (in total 1550 plots) (Ulrich et al., 2021). For each cluster, we started SAD fitting with a randomly chosen plot of at least 20 species and gradually added the cover values of all other plots in random order to obtain a cumulative plot sequence (CPS), which also reflects increasing sample area. This additive process implies that cumulative cover values might be larger than 100. We note that these CPSs do not form sequences of spatially continuous vegetation but are aggregations of discontinuous plots. Of course, the SAD at the starting point and the specific ordering of plots during accumulation might influence the inferred scaling behavior and increase the variance in scaling patterns across these CPSs. However, we did not average values of several runs of random accumulation within each plot series because such averaging would artificially smooth the spatial scaling and bias the pattern toward what is predicted from sample theory in homogeneous environments. The high number of individual plots within each CPS guaranteed that the assessment of changes in SAD parameters across a CPS would not be influenced by the ordering of plot combination.

For each individual plot and each accumulation step of the CPS, we fitted the Weibull distribution to the species rank–ln‐abundance distribution (Whittaker representation) (Whittaker, 1965) using ln‐transformed relative cover values according to standard practice. The final step of each CPS provides a rough estimate of the abundance distribution of the regional species pool for that cluster. The complete data set, including fitted parameters and moments of the SAD distributions, is contained in Ulrich et al. (2021).

### Environmental variables

The GrassPlot data set contains a range of environmental and geographical variables known to be important drivers of plant diversity and distributions; certain variables are only available for a subset of plots. In this study, for all plots we used the geographical variables latitude, longitude, and elevation. For 1111 plots, information on mean soil depth, for 569 plots information on soil organic matter content (OMC), and for 338 plots information on soil C/N ratio was available. Additionally, we retrieved data for average annual temperature, annual precipitation, temperature range and precipitation variability for all plots from the CHELSA climate database (Karger et al., 2016). The complete geographical and environmental raw data for each plot are contained in Ulrich et al. (2021).

### Data analysis

Prior work on the variation in SAD shape between sites largely relied on comparing the fits of different standard models. However, reliable model comparisons need large sample sizes, at least on the order of 20 species (Ulrich et al., 2010; Wilson, 1993). Therefore, here we take a two‐pronged approach. First, we rely on model‐independent moments of SADs: the variance (σ^2^, second moment) as a measure of the range in plant cover, the skewness (γ, third moment) as a measure of an excess of relatively rare or abundant species, and the kurtosis (δ, fourth moment) as a quantification of the proportion of species with relatively intermediate cover. Additionally, we fitted the two‐parameter Weibull distribution to the observed plant cover data. Recently, Ulrich et al. (2018, 2020) demonstrated that this distribution is sufficiently flexible to mimic a wide range of observed SAD shapes. The model allows for a continuous tracing of the changes in the two Weibull parameter values (scale and shape) in order to assess the scaling properties of observed SADs and to relate these to environmental correlates.

#### Fitting the Weibull model to empirical SADs

**FIGURE 1:**
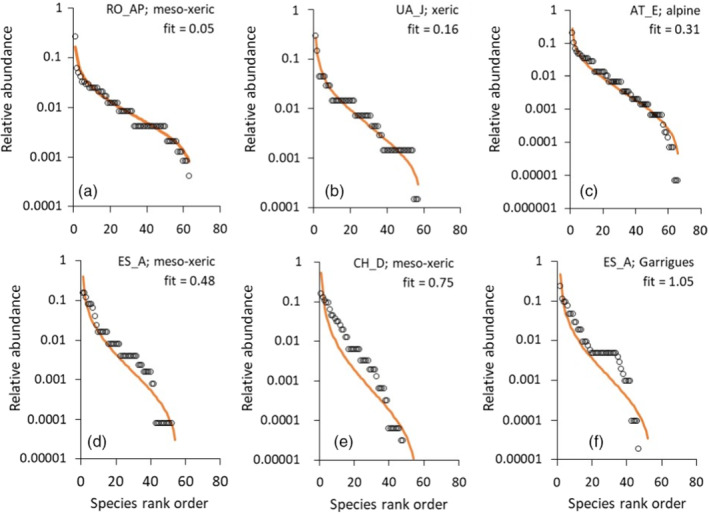
Six example fits of the Weibull distribution to grassland community species abundance distributions (SADs), with different values of the goodness‐of‐fit measure. Goodness of fit was calculated as the average sum of least squares: $\mathrm{fit}=\frac{1}{S}\sum_{1}^{S}{\left(\ln\;p_{i}-\ln\;w_{j}\right)}^{2}$, where ln *p*_*i*_ and ln *w*_*j*_ denote ln‐transformed observed and Weibull fitted relative percentage cover values. Letters (e.g., RO_AP) refer to CPS plot code (Appendix S1: Table S5) (Ulrich et al., 2021)

The two‐parameter form of the Weibull distribution has the probability density function (PDF)

(1) $$p\left(x>0;\varphi ;\lambda \right)=\frac{\varphi }{\lambda }{\left(\frac{x}{\lambda }\right)}^{\varphi -1}e^{-{\left(\frac{x}{\lambda }\right)}^{\varphi }}$$

where φ is the shape and λ the scale parameter. The Weibull shape parameter (φ) decreases with increasing skewness of the distribution, and the scale parameter (λ) increases with the observed range in abundance (Ulrich et al., 2018). Consequently, λ and σ^2^ are positively correlated (present data: *r* = 0.73). The φ/λ quotient is more closely related to the empirical variance of the SAD by a power function (cf. Appendix S1: Figure S2 for the present data set). Shape parameters around φ = 2 mimic lognormal distributions, whereas φ = 1 refers approximately to log‐series distributions. When applied to species abundances, the random variate *x* must contain log‐transformed abundances calculated for all species (S). The Fortran code used for asymptotic ordinary least‐squares fitting of the Weibull distribution (using a pattern‐seeking algorithm) has already been presented in Ulrich et al. (2018) and is freely available from the corresponding author upon request. As a measure of goodness of fit we used the average sum of least squares: $\mathrm{fit}=\frac{1}{S}\sum_{1}^{S}{\left(\ln\;p_{i}-\ln\;w_{j}\right)}^{2}$,where ln *p*_*i*_ and ln *w*_*j*_ denote the ln‐transformed observed and Weibull fitted relative abundances, respectively. Ulrich et al. (2018) compared different types of SAD from small to intermediate sized Japanese forest tree communities (<100 species) and reported fit values <0.05 as being excellent, whereas fit values >0.3 were considered poor. Figure 1 contains six typical examples from the present data set of excellent to poor fits and demonstrates that fit values <0.3 can still be considered very good. Weibull model fits to the SADs of each plot, including observed and estimated cover values for each species together with respective SDs of the estimates, are contained in Ulrich et al. (2021).

### Statistical analysis

For each of the individual plots and each accumulation step of the CPSs, we calculated the skewness (γ) and the kurtosis (δ) of the SAD. We note that a symmetric lognormal distribution has a skewness of γ = 0, while a negative skewness indicates an excess of relatively rare species (note that this regards log‐abundance distributions: distributions of raw abundances with an excess of rare species are right skewed) (e.g., Šizling et al., 2009). A standard lognormal distribution is characterized by a kurtosis of δ = 3. Higher kurtosis values mark an excess of species with intermediate abundances. Additionally, we calculated the proportional β‐diversity of each cumulative plot series as β=1−α/γ, where α is the average local (plot) and γ the total species richness of the CPS.

**FIGURE 2:**
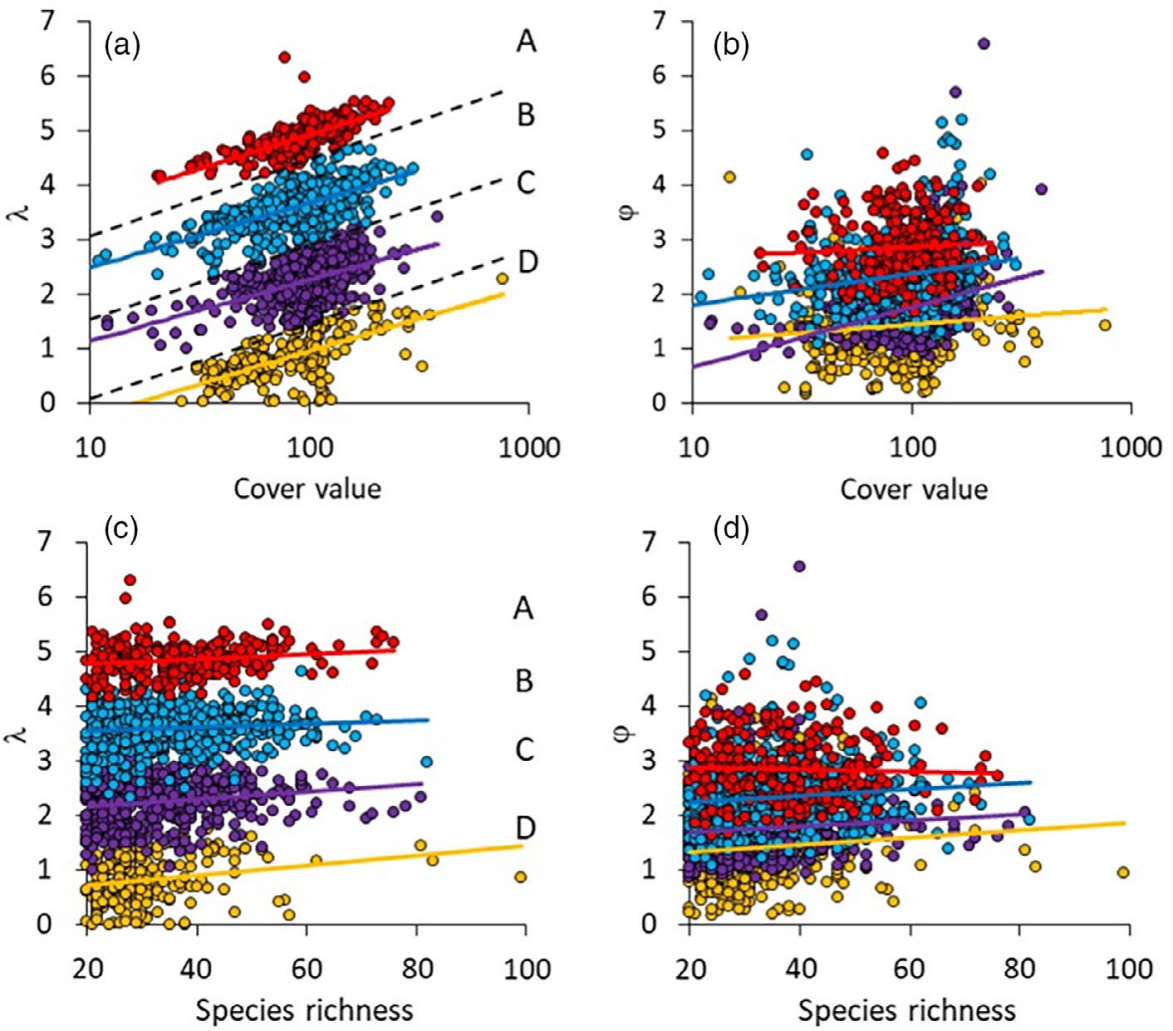
Plots of (a, c) Weibull scale parameter λ and (b, d) shape parameter φ against (a, b) total plant cover values and (c, d) species richness for all 1725 single plots returned four clearly defined groups of plots (A, B, C, D) with respect to the intercept value of λ. Groups were less clearly defined with respect to φ. Community membership of these four groups is provided in Ulrich et al. (2021). Regression lines refer to (a, b) ordinary least‐squares logarithmic and (c, d) linear regressions. Broken lines in (a) indicate boundaries of group membership

**FIGURE 3:**
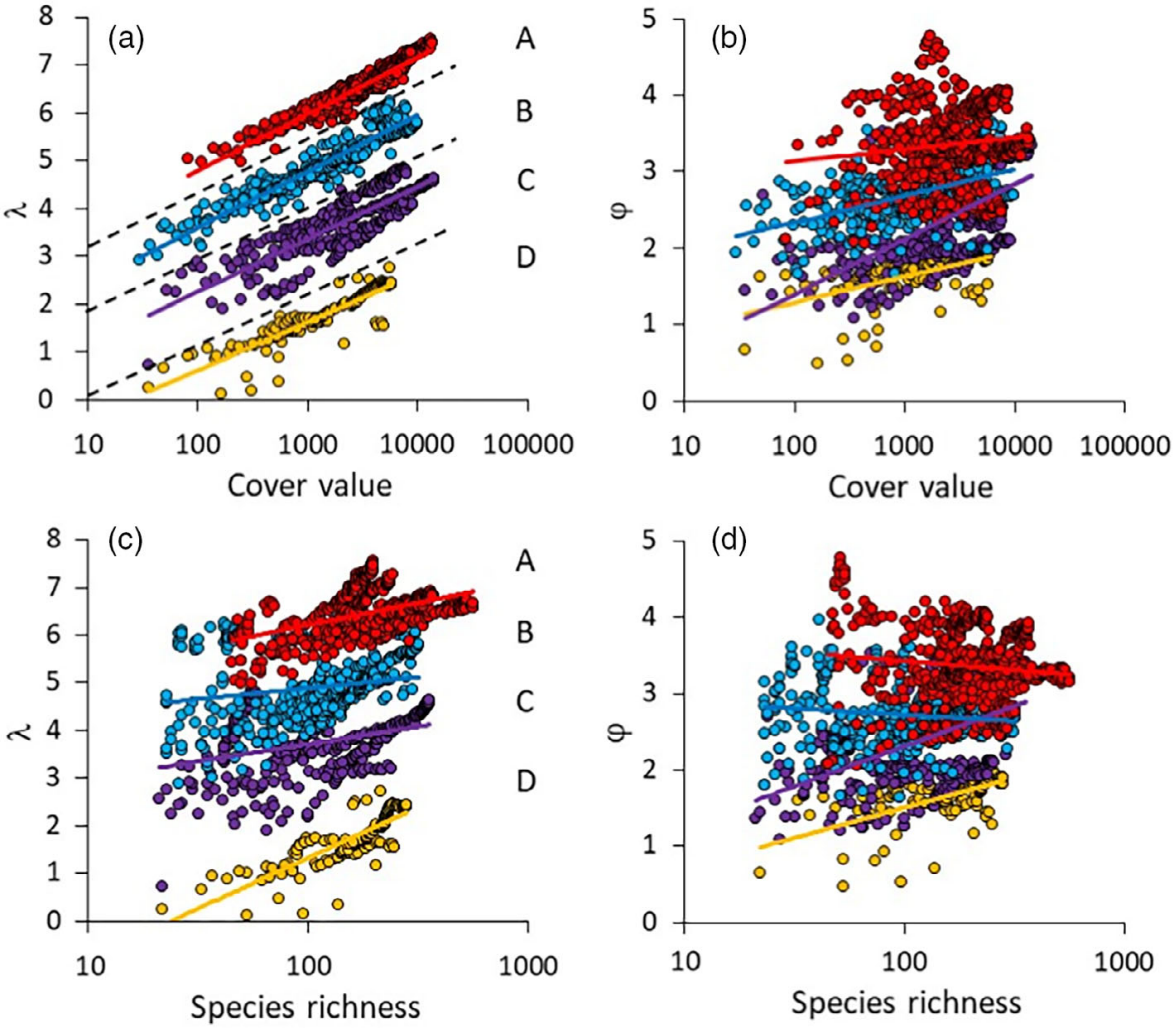
Plots of (a, c) Weibull fit scale parameter λ and (b, d) shape parameter φ against (a, b) plant cumulative cover and (c, d) species richness for all aggregation steps of cumulative plot series highlight the four clearly defined groups of plots (A, B, C, D) with respect to the intercept value of λ. Groups were less clearly defined with respect to φ. Community membership of these four groups is provided in Ulrich et al. (2021). Regression lines refer to ordinary least‐squares logarithmic regressions. Broken lines in (a) indicate boundaries of group membership

Graphical comparisons of λ‐values of single plots (Figure 2a) and CPS (Figure 3a) against total cover indicated the existence of four clearly separated groups of SADs. We optimized classification using k‐means clustering applied to the quotient of λ/ln(cover values) and fitted ordinary logarithmic least‐squares regressions to each group individually. Discriminant analysis served to relate these groups to environmental variables. Nested linear mixed‐effects modeling (GLM) and parametric ANOVA with post hoc Tukey tests were used to relate the SAD parameters to community species richness, total ln‐abundance, and environmental variables. To infer any nonlinear patterns with regard to changes in the various SAD moments and parameters (γ, δ, φ, λ) with spatial scale, we included the squared zero centered ln‐cover term (separately calculated for each of the 40 CPSs into the analysis). Because the spatial extent of the study area, that is, the area encompassed by the plots within the CPSs, might influence the results we also added the average pairwise plot distance within each CPS as a covariate. We estimated the impacts of predictor variables from partial $\eta^{2}$ values,

$\text{partial}\;\eta^{2}=\frac{{\mathrm{SS}}_{\text{effect}}}{{\mathrm{SS}}_{\text{effect}}+{\mathrm{SS}}_{\text{error}}}$,

where SS denotes the sum of squares. Calculations were undertaken using Statistica 12.0.

We also applied polynomial ordinary linear segmented least‐squares regression to the SAD parameter values versus ln‐cover of each of the 40 CPSs, as implemented in SigmaPlot 14. A significant breakpoint indicates a nonlinear scaling of the respective parameters along the total cover value (and therefore area) axis of a given CPS. As the spatial distribution of the analyzed GrassPlot plots was clustered and absolute spatial distances might be important, we used eigenvector mapping and added the dominant eigenvector (EV1) of the Euclidean plot distance matrix to the GLM models as a covariate.

## RESULTS

### Goodness of fit

The majority of the grassland plant SADs were very well fitted by the Weibull distribution (fits in Ulrich et al., 2021, Figure 1; Appendix S1: Figure S3). Among the 1725 individual plots, 400 (23.2%) had fit values <0.1, indicating excellent to very good fits (e.g., Figure 1a), 1135 plots (65.8%) had fit values <0.3, indicating very good fits (e.g., Figures 1b, c), and only 196 (11.4%) were comparatively weakly fitted by the Weibull distribution (fit >0.75, e.g., Figure 1e,f). Goodness of fit differed significantly between plots (Appendix S1: Table S1). Species richness did not significantly influence goodness of fit (Appendix S1: Table S1).

### Variability in SAD parameters between SAD groups

With 868 individual plots (50.3%) and 1263 CPS aggregation steps (81.5%), most fitted SADs were characterized by φ > 2.0, equivalent to lognormal‐type SADs. Only 423 individual plots (24.5%) and 47 of the CPS aggregation steps (3.0%) had φ < 1.5, equivalent to a log‐series SAD. SAD skewness γ decreased with plot abundance, indicating an excess of rare species at higher total cover values (Appendix S1: Figure S4c). Kurtosis δ was largely independent of cover and richness (Appendix S1: Figure S4d,h).

**FIGURE 4:**
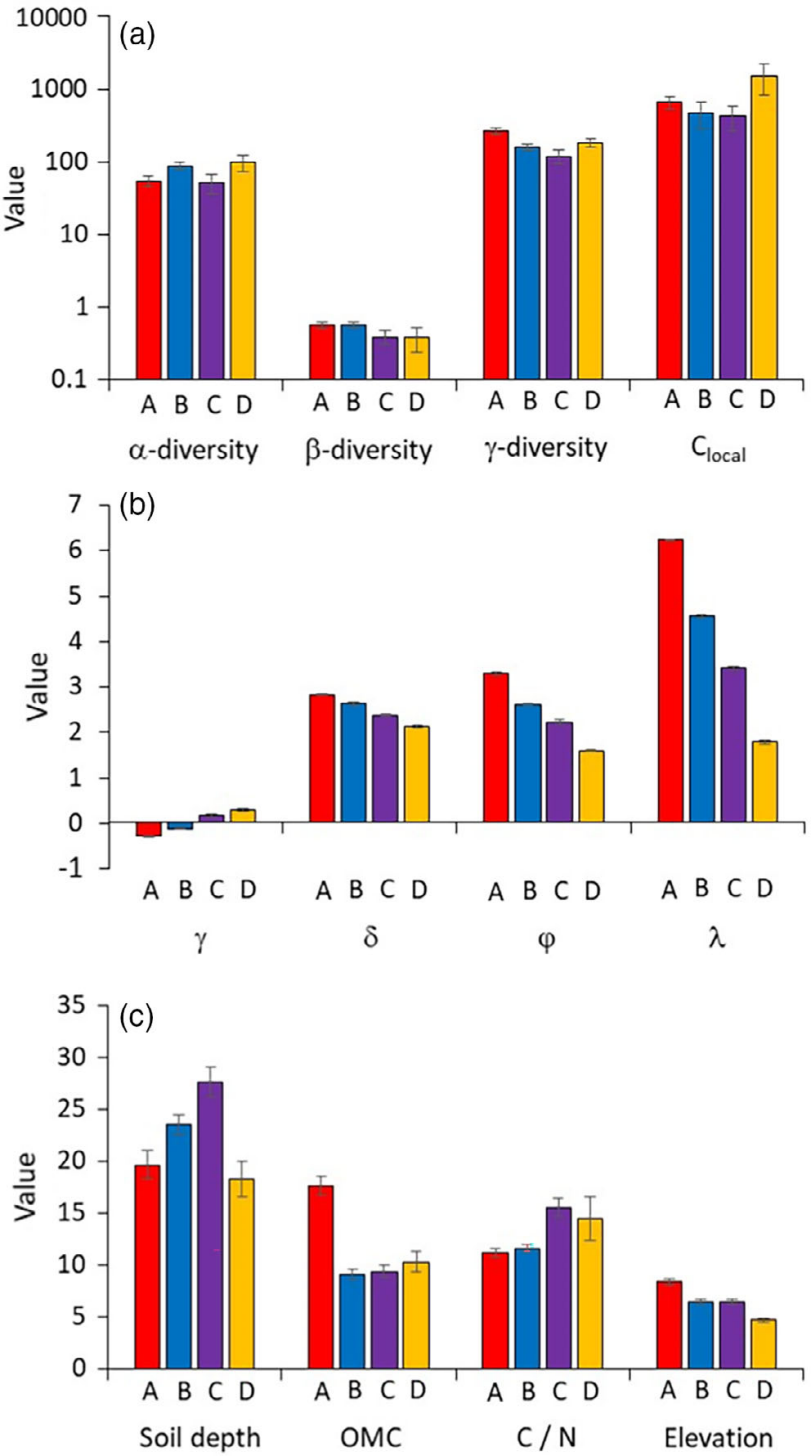
(a) Average values of α‐, β‐, and γ‐diversity, and average local cover values (*C*_local_) for cumulative plot sequences (CPS) within the four species abundance distribution (SAD) groups (uppercase letters) identified in Figure 2. There were no significant differences between types. (b) Average values of skewness (γ), kurtosis (δ), shape (φ), and scale (λ) parameters of single grassland SADs, within the four groups. All group comparisons significantly differed within each parameter. (c) Average soil depth, soil organic matter content (OMC), soil C/N ratios, and plot elevation within the four groups. For soil depth, Groups B and C significantly differed from Groups A and B; for OMC Group A significantly differed from B, C, and D; for C/N ratios C and D differed from A and B; and for elevation A and D differed and both differed from B and C. Error bars denote SEs. Significances at the two‐sided 1% error level between groups were tested with one‐way ANOVA and post hoc Tukey tests

Plots of φ and λ against cover values and species richness in combination with k‐means cluster analysis pointed to four distinct groups of grassland SADs differentiated by the scaling of λ with plant cover (Figures 2a and 3a; Appendix S1: Table S2). Group differentiation was less obvious with respect to φ (Figures 2 and 3; Appendix S1: Table S2), although k‐means clustering still confirmed >40.0% group memberships (Figures 2b,d and 3b,d; Appendix S1: Table S2). The four groups did not significantly differ with respect to α‐, β‐, and γ‐diversity or to local cover (Figure 4a). One‐way ANOVA indicated that there was a moderate effect of vegetation type on group membership (partial $\eta^{2}$ = 0.08, *p* < 0.001). In particular, Group B dominated in alpine, xeric, rocky, and sandy dry grassland communities, while Group C dominated in meso‐xeric, mesic, and Mediterranean grasslands, as well as in wetlands (Appendix S1: Figure S3a).

Linear modeling detected significant differences among the four SAD groups with respect to the γ and δ of the SAD distribution and the Weibull parameter values (Table 1, Figure 4). Groups A and B SADs were on average characterized by a slightly negative empirical γ, indicating an excess of rare species (Figure 4b), while the SADs of Groups C and D were significantly right skewed in accordance with an excess of abundant species (Figure 4b). For all four groups, δ ranged between 2 and 3, with a decrease toward Group D (Figure 4b). Weibull φ was lowest (<2.0) for Group D communities (Figure 4b).

**TABLE 1:** General linear modeling detected significant differences in empirical species abundance distribution (SAD) skewness (γ) and kurtosis (δ), and Weibull shape (φ) and scale (λ) parameters, between four groups of grassland plant communities (as defined in Figure 2) and between vegetation types

Variable	df	γ		δ		φ		λ	
		Partial $\eta^{2}$	β	Partial $\eta^{2}$	β	Partial $\eta^{2}$	β	Partial $\eta^{2}$	β
SAD group	3	0.29***	…	0.04***	…	0.36***	…	0.94***	…
Vegetation type	18	0.03***	…	0.05***	…	0.02***	…	0.04***	…
*T*_mean_	1	0.02***	−0.24	0.01**	0.23	0.03***	0.32	<0.01	−0.01
*T*_range_	1	<0.01	0.05	0.01**	−0.20	0.01**	−0.13	0.01**	0.04
*P*_mean_	1	<0.01	0.06	<0.01	0.00	<0.01	−0.03	<0.01	0.00
*P*_seasonality_	1	0.02***	0.28	0.01**	−0.18	0.04***	−0.33	<0.01	−0.02
EV1	1	<0.01	0.02	<0.01	−0.03	<0.01	0.02	<0.01	0.02
S	1	<0.01	−0.01	<0.01	0.02	<0.01	0.04	0.01**	−0.02
ln C	1	0.04***	−0.20	<0.01	−0.04	0.03***	0.17	0.34***	0.20
*r* ^2^	…	0.39***	…	0.10***	…	0.44***	…	0.95***	…

*Note*: *T*_mean_, *P*_mean_, annual mean temperature and precipitation; *T*_range_, temperature range; *P*_seasonality_, precipitation seasonality; ln C, ln‐transformed cover values; S, species richness; and the dominant eigenvector of plot spatial distances (EV1) served as metric covariates. Vegetation type entered the model as a random effect. Partial $\eta^{2}$‐ and β‐values are shown. Parametric significances: **p < 0.01, ***p < 0.001. *N* = 1719 for all four models.

### Environmental influences

The climatic variables used here, in addition to elevation and latitude, did not significantly influence the observed SAD shapes (Table 1; Appendix S1: Figure S5). φ and λ increased and γ decreased with soil OMC (Appendix S1: Figure S6e,g,h), while γ increased and λ decreased with soil C/N ratio (Appendix S1: Figure S6i,l). Discriminant analysis also did not detect any significant influence of climatic variables on SAD group membership (Appendix S1: Table S3). However, we found a strong indirect influence of soil characteristics on SAD group membership and therefore on SAD shape (Figure 4c; Appendix S1: Table S3). Group C communities were related to deeper soils with increased C/N ratios, while increased OMC was most common for Group A communities (Figure 4c). Group A communities dominated at higher, and Group D communities at lower, elevation (Figure 4c).

### Spatial scaling of SAD parameters

The Weibull parameters increased with increasing cumulative cover values (equivalent to increasing area) in a CPS‐specific manner within and among each of the four groups (Figure 3). A hierarchical GLM detected highly significant influences of group membership (largely congruent with geographic position), vegetation type, and cumulative cover on the SAD moments and Weibull parameters (Table 3). The latter effect was not visible when single plots were used (Appendix S1: Table S4). The spatial distances of plots within each CPS had only a minor influence on parameter variation (Table 3).

**FIGURE 5:**
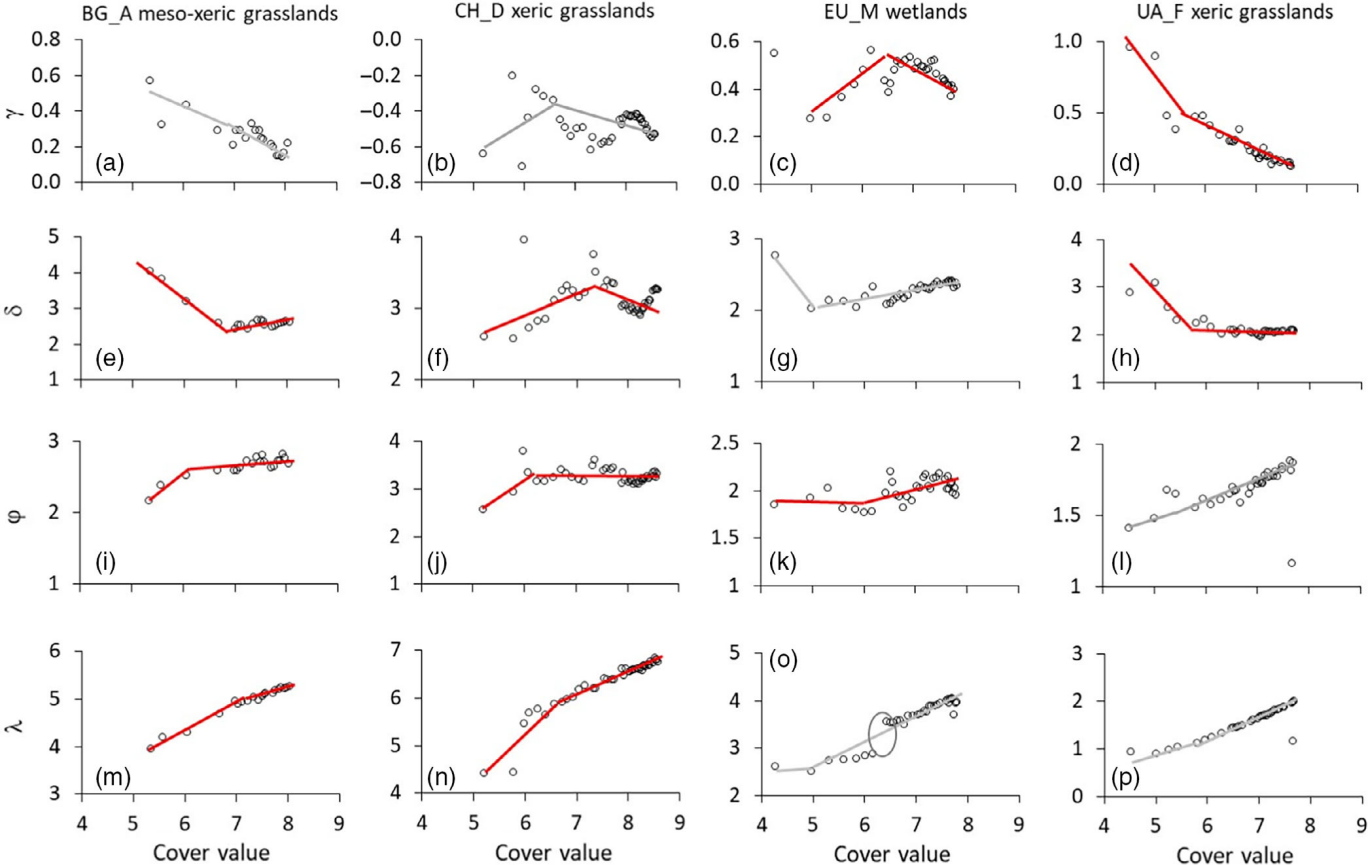
Plots of the relationships between species abundance distribution (SAD) skewness (γ), kurtosis (δ), Weibull fit shape (φ), and scale parameters (λ), and ln‐transformed cover values for four representative cumulative plot series. Red regression lines show significant (*p* < 0.001) and gray lines nonsignificant (*p* > 0.10) piecewise regressions. The oval in (o) denotes a step change in scale value. Site information for each of the four sites can be found in Ulrich et al. (2021).

Segmented linear regression of ln‐transformed cover values against γ, δ, φ, and λ, within each CPS, revealed highly variable nonlinear and often irregular relationships and, in 69% of the CPSs, significant breakpoints (Figure 5, Table 3; Appendix S1: Table S5). In 47% of the CPSs, the studied parameters decreased or increased continuously with increasing cover values (Appendix S1: Table S5). Frequently, parameter values peaked at intermediate cumulative cover values (γ: 27.5%, δ: 45.0%, φ: 40.0%, λ 7.5%) (Appendix S1: Table S5). Patterns of parameter change were generally not consistent within each CPS (Figure 5; Appendix S1: Table S5).

## DISCUSSION

### Model fits and Weibull parameters

Our study shows that the two‐parameter Weibull distribution generally provides excellent fits to cover‐based plant community SAD data (Figure 1; Appendix S1: Figure S3). This is in agreement with previous work that found the model did a good job of mimicking the distributions of Japanese woody plant communities (Ulrich et al., 2018, 2020). Together with these previous studies, our results indicate that the Weibull model provides a useful tool for researchers interested in SADs and their ecological implications. Based on the Weibull fits, we detected four different types of local communities characterized by different φ and λ parameter combinations. These types were not directly linked to differences in richness, abundance, and environmental conditions. It is worth highlighting that these four groups might not have been detected in SAD analyses based on classical fits to species number–log abundance distributions (Preston representations, Matthews & Whittaker, 2014) or in analyses based solely on the moments of distributions or model goodness of fit (e.g., Gross et al., 2017; Ulrich et al., 2010).

In answer to our first question, what types of SADs are realized for vascular plants in extra‐tropical grasslands, we found that in more than half of all single communities, particularly Group A and B communities, and in more than two‐thirds of CPSs, φ was greater than 2, indicating lognormal‐type abundance distributions across the spatial scales examined (Figure 3). These results are in line with Ulrich et al. (2016), who reported a similar prevalence of lognormal distributions in global dryland plant communities. Lognormal SADs are generally considered to be characteristic of closed, dispersal‐limited local communities that are strongly controlled by biotic interactions (Hubbell, 2001; Magurran & Henderson, 2003; Ulrich et al., 2010), although Connolly et al. (2005), Enquist et al. (2019), and Antão et al. (2021) found evidence that plant and animal communities also exhibited lognormal SADs at large spatial scales. With respect to grassland plants, relative abundances at the local scale are generally thought to be mainly determined by life form and, therefore, by habitat‐filtering mechanisms and competitive interactions, not by dispersal (Eriksson & Jakobsson, 1998). Silva et al. (2010) and Wu et al. (2019), who analyzed SADs of sample distributions in Brazilian Cerrado grasslands and Chinese forest trees, respectively, indicated that regional understory forest plant SADs are of the log‐series type, as assumed by dispersal‐driven ecological drift models (Hubbell, 2001). Our results contrast with these findings. We note that the cumulative areas of the CPSs considered here are much smaller than those of Silva et al. (2010) and Wu et al. (2019), which may explain these differences. Nonetheless, our results do not provide support for ecological theories based on the assumption of local and regional‐scale log‐series SADs, such as the maximum entropy theory of ecology (Harte, 2011), although we cannot exclude the possibility that larger, continental grassland species pools might still be log‐series distributed, while at intermediate scales lognormal‐type distributions prevail.

There is an ongoing debate on whether the analysis of SADs can reveal the underlying processes of community assembly (e.g., Matthews & Whittaker, 2015; Zhou & Ning, 2017; Wang et al., 2018; Feng et al. 2021). This debate has two distinct aspects that are often confused. The first aspect regards the type of SAD model or, more precisely, whether process‐orientated niche division (e.g., Tokeshi 1998) or neutral models (Fisher et al., 1943; Harte, 2011) provide better representations of nature. This question is now arguably answered—both types of models can mimic observed SADs, making it impossible to infer the processes behind community assembly from model fitting alone. Consequently, and based on the application of Occam's razor, probabilistic distribution–based models, like the Weibull model, that make no explicit assumptions about ecological processes are typically preferred (Hubbell, 2001; Locey & White, 2013; Preston, 1948; Ulrich et al., 2018). The second aspect concerns the question of whether the observed parameter values of well‐fitting SAD models tell us something about the processes of community assembly and whether they can be related to ecological factors. For example, previous studies at coarse spatial scales found SAD shape to be partly driven by variation in climatic factors (e.g., Matthews et al., 2019; Ulrich et al., 2016). Our results indicate that there are strong constraints on the Weibull parameter space with respect to SAD shape but to a lesser extent with respect to SAD scale (Figure 4b). Put another way, observed SADs are characterized more or less by symmetric distributions (low skewness) but with highly variable absolute differences in abundances between species (Figure 4). Symmetric lognormal‐type SAD distributions imply no excess of highly abundant or rare species. Such an excess has been found in many animal and plant SADs, particularly when using heterogeneous samples across local habitat boundaries, where dispersal might play a major role (Enquist et al., 2019; McGill, 2003). In plant communities, dispersal dominates at early successional stages while species interactions increase in importance toward climax communities (Makoto & Wilson, 2018). Although we did not quantify the compositional dynamics of the grassland communities considered in this study, we assume that the majority are comparatively stable and hypothesize that compositional stability in plant communities is linked with more or less equal numbers of very abundant and very rare species.

Ulrich et al. (2018) related the λ (Weibull scale) parameter to the magnitude of abundance differences within a community and, therefore, to the variance (σ^2^) of the SAD. This work, in combination with our results (Appendix S1: Figure S2), indicates that σ^2^ and λ are not linked by a simple positive relationship, so each quantifies a different aspect of the SAD. More precisely, σ^2^ is a power function of the φ/λ relationship, with a sharp lower boundary reflecting minimum possible values of φ/λ approaching a lower boundary of ~0.25 at maximum observed variance in abundance (σ^2^ ≈ 16) (Appendix S1: Figure S2). Ulrich et al. (2020) reported the same lower value of φ/λ ≈ 0.25 for Japanese woody plants. Importantly, this boundary is not set by the internal mathematical properties of the distribution. Therefore, we speculate that this boundary represents abundance limits for ecologically stable local plant communities.

The kurtosis δ is a widely, although wrongly, neglected moment of SADs (Gross et al., 2017). Despite the discussion on the correct statistical interpretation of this fourth moment (DeCarlo, 1997), with respect to SADs we can safely argue that a high kurtosis is linked to an excess of species with intermediate abundance. Low δ, in contrast, points to a lack of these middle‐class species. These are subdominants that might gain dominance either by competitive effects or after disturbances (Ulrich et al., 2016). Our results do not point to an excess of such subdominants (Figure 4). Except for communities with very low abundance differences, we found a median value of δ = 2.64 (quartiles: 2.20 and 3.26) in line with distributions narrower than predicted by the standard lognormal (δ = 3). Unfortunately, most prior work on SADs did not report δ‐values. A reassessment of data provided by Ulrich et al. (2020) and Gross et al. (2017) returned a median kurtosis of δ = 2.47 (2.11–3.06) for Japanese woody plant communities and δ = 2.45 (1.99–3.09) for global dryland plants. With respect to the SADs of woody plants, birds, mammals, and miscellaneous other taxa, a recent meta‐analysis also revealed a general tendency for increased proportions of rare species and, consequently, a lower proportion of species with intermediate abundance (Diaz et al., 2021). We interpret this general tendency for local plant communities to exhibit a deficit of subdominants in terms of competitive exclusion along the hierarchy of competitive strength. However, this is an ad hoc assumption that needs to be backed up by corresponding simulation studies and field observations in hierarchical (transitive) and intransitive competitive networks of plant species (cf. Soliveres et al. 2015; Wang et al., 2018). Importantly, our argument is based on a comparison with the standard lognormal distribution that serves as a random expectation. Neutral local communities with reduced dispersal ability will be close to this assumption (Hubbell, 2001). Nevertheless, a more appropriate null standard is required to enable better interpretation of SAD kurtosis values.

A feature of this study concerns the use of cover data as opposed to the actual numbers of individuals. However, while the number of individuals is the classical abundance metric in SAD studies, other measures are also used (e.g., biomass, cover), particularly in plant studies where the counting of individuals in most cases is infeasible because the majority of species exhibit clonal growth. Anderson et al. (2012) demonstrated how different measures of plant abundance influence the assessment of the SAD. Of course, species log cover–rank order SADs (Whittaker representations) as used here will give different dominance orders than count data, simply owing to the much larger variance of count data and the resultant higher scale parameters of the respective model distributions (Ferreira de Lima et al., 2020). Clearly, Whittaker representations are least affected by this categorization affect. Nevertheless, the metric used to quantify abundance might influence, for instance, the assessment of the proportion of very abundant or rare species and might affect comparisons of SADs, for instance in meta‐analyses, based on different types of binning. Here we used the same abundance cover quantification within a 0–100 scale for all plots. Therefore, the data and, thus, the distribution and model parameters should be comparable. However, caution should be applied when comparing the parameter values of this study with those from other studies using different abundance measures.

### Environmental correlates

With regard to our second question of whether SAD shape changes across environmental gradients across the Palaearctic, we found evidence supporting the hypothesis that environmental conditions (climate and soil) influence the shape of SADs of grassland plant communities at local scales, to some extent (Tables 1 and 2, Figure 4; Appendix S1: Figures S3, S5, and S6). For example, we found a strong signal that deeper soils were associated with Group B and C SADs characterized by intermediate Weibull parameters and symmetric distributions without an excess of either rare or abundance species (Figure 4). Group A communities were associated with richer soils with higher OMC and low C/N ratios, as well as high φ‐values and an excess of rare species (Figure 4). Additionally, our results indicate that such soil conditions might also favor weaker local competitors, leading to a shallower dominance hierarchy and increased proportions of subdominants (Figure 4). This finding somewhat contradicts Feng et al. (2021), who reported that increased soil nutrient concentrations caused decreased taxonomic diversity of forest tree species and increased species dominance and rarity. Prior studies indeed suggested that higher amounts of resources allow for a wider range of species to enter local communities, making their assembly more colonization driven and leading to log‐series SADs with an excess of rare species (see also Magurran & Henderson 2003; Ulrich et al. 2010). Additionally, Ulrich et al. (2016) found that dryland plant communities on poorer soils strongly filter for specialist species, whereas those species for which such soils are suboptimal are excluded; these processes then drive SADs toward lognormal shapes. These contrasting findings caution against simple generalizations. It is possible that, in grasslands, interaction‐driven lognormal type SADs tend to prevail within a wider range of environmental conditions, including more extreme habitats like arid environments, while log‐series distributions are mainly restricted to comparatively species‐rich communities on humid and fertile soils.

**TABLE 2:** General linear modeling detected significant differences in empirical species abundance distribution (SAD) skewness (γ) and kurtosis (δ), and Weibull shape (φ) and scale (λ) parameters, between four groups of grassland plant communities (as defined in Figure 2) and between vegetation types

Variable	df	γ		δ		φ		λ	
		Partial $\eta^{2}$	β	Partial $\eta^{2}$	β	Partial $\eta^{2}$	β	Partial $\eta^{2}$	β
SAD group	3	0.14***	…	0.01	…	0.23***	…	0.89***	…
Vegetation type	10	0.04	…	0.02	…	0.04	…	0.05*	…
Soil depth	1	0.01*	0.13	<0.01	0.04	<0.01	−0.06	<0.01	<0.01
Organic matter content	1	<0.01	−0.02	0.01	−0.13	<0.01	0.02	<0.01	−0.02
EV1	1	<0.01	0.08	<0.01	<0.01	<0.01	0.06	<0.01	−0.02
S	1	0.01	0.08	<0.01	−0.02	<0.01	−0.04	0.06***	−0.08
ln C	1	0.01*	−0.12	<0.01	−0.01	0.02**	0.16	0.46***	0.30
Elevation	1	<0.01	0.04	<0.01	−0.02	<0.01	−0.05	0.01*	0.04
*r* ^2^	…	0.26***	…	<0.01	…	0.32***	…	0.94***	…

*Note*: ln C, ln‐transformed cover values; S, species richness; and the dominant eigenvector of plot spatial distances (EV1) served as metric covariates. The C/N ratio was not included because of the low number of remaining data points. Vegetation type entered the model as a random effect. Partial $\eta^{2}$ effect sizes and standardized slope parameters (*β*‐values) are shown. Parametric significances: **p < 0.01, ***p < 0.001. *N* = 442 plots for which all soil data were available.

**TABLE 3:** Hierarchically nested general linear modeling (vegetation type nested in cumulative plot sequence [CPS], Z‐transformed cover values *Z*_*C*_, separately calculated for each CPS, and squared cover values *Z*_*C*_^2^ nested in vegetation type) detected significant nonlinear dependencies of empirical species abundance distribution (SAD) skewness (γ) and kurtosis (δ), and Weibull shape (φ) and scale (λ) parameters, with increasing ln‐transformed cover values within each CPS

Variable	Nested in	df	γ	δ	φ	λ
CPS		23	0.74***	0.69***	0.81***	0.92***
Vegetation type	CPS	5	0.07***	0.14***	0.17***	0.29***
Z_C_	Vegetation type	8	0.08***	0.06***	0.17***	0.42***
Z_C_^2^	Vegetation type	8	0.04***	0.02**	0.04***	0.05***
Distance		1	<0.001	0.02***	0.02***	<0.001
*r* ^2^		…	0.78***	0.74***	0.87***	0.94***

*Note*: CPS and vegetation type entered the model as random effects. Given are partial $\eta^{2}$ effect sizes. Parametric significances: **p < 0.01***, *p* < 0.001. The average distance of plots within each CPS served as covariate. *N* = 1550 for all four models.

### Spatial scaling

Finally, we asked whether local SAD parameters were scale‐invariant or changed predictably with increasing spatial scale. Such changes are predicted by sample theory models (Green & Plotkin, 2007; Locey & White, 2013), although the precise patterns of change are unknown because, for the lognormal and the Weibull distributions, no scaling equations exist.

The scaling of SADs is not necessarily a gradual linear process (Rosindell & Cornell, 2013). Below the interaction neighborhoods of individual plants (sensu Addicott et al., 1987), SADs are expected to be driven by competitive and facilitation effects (Callaway & Walker, 1997). Filter effects drive SADs above and below these neighborhoods. With increasing spatial scale, stochastic processes due to colonization/extinction dynamics take over (Ferreira de Lima et al., 2020). This discontinuity of ecological processes strongly indicates the existence of scaling regions of SADs and, possibly, scale‐specific SAD shapes. Thus, we hypothesized that the detection of such scaling regions should be an indicator of changes in the dominance of ecological processes and, consequently, in the hierarchy of dominance in abundance. The scaling of SAD parameters should therefore provide information about the (dynamic) boundaries of interaction‐shaped local plant communities.

We were surprised to see that the parameters analyzed here often did not change gradually with increasing spatial scale (Figure 5; Appendix S1: Table S5), as predicted by sample theory models (Green & Plotkin, 2007; Hubbell, 2001; Locey & White, 2013). Moreover, the signs of the segment slopes were not consistent within study sites and vegetation types (Figure 5; Appendix S1: Table S5), indicating that SAD scaling is very location‐specific. The comparatively weak effects of climatic and soil covariates on SAD parameters reported here point to local stochastic dynamics rather than SADs in Palaearctic grasslands being shaped by niche and environmental drivers. Sampling models do not consider this specificity and are only based on general assumptions about the spatial distribution of species that determine the accumulation of new species with increasing sample space. In particular, they assume that newly sampled species at higher sample sizes should be comparatively rare. However, our results are in line with a pattern where stronger species aggregation, but also environmental heterogeneity, might cause the newly sampled species at larger sample sizes to account for comparatively higher proportions of total abundance. This would result in a shift toward higher δ‐ and φ‐values as sample size increases. Future studies need to incorporate such data in order to develop a precise SAD spatial scaling framework that provides insight into the dependency of SAD scaling on the pattern of plant species aggregation.

## AUTHOR CONTRIBUTIONS

Werner Ulrich conceived the idea of this paper and wrote the first draft. Most authors contributed data, while Jürgen Dengler served as custodian and Idoia Biurrun as database manager of the GrassPlot database. Idoia Biurrun extracted the appropriate data from the GrassPlot database. Werner Ulrich conducted the statistical analyses, and Thomas J. Matthews contributed figures and conceptual and analytical input. Jürgen Dengler provided methodological and botanical input. All authors checked, improved, and approved the manuscript.

## CONFLICT OF INTEREST

The authors declare no conflict of interest.

## Supporting information


Appendix S1

## Data Availability

Data (Ulrich et al., 2021) are available in Figshare at https://doi.org/10.6084/m9.figshare.16870641. Vegetation‐plot data were obtained from the GrassPlot database; our data are a subset of the GrassPlot data suited for abundance analyses, with these data completely contained in our Figshare data set.
