# Supplementary material for: Environmental drivers and spatial scaling of species abundance distributions in Palaearctic grassland vegetation
Source: Ecology. 2022 May 28;103(8):e3725. doi: 10.1002/ecy.3725 (PMC9540260; doi:10.1002/ecy.3725)

Appendix S1

Ecology

Environmental drivers and spatial scaling of species abundance distributions in Palaearctic grassland vegetation

Werner Ulrich, Thomas J. Matthews, Idoia Biurrun, Juan Antonio Campos, Patryk Czortek, Iwona Dembicz, Franz Essl, Goffredo Filibeck, Gian-Pietro Giusso del Galdo, Behlül Güler, Alireza Naqinezhad, Péter Török, Jürgen Dengler

Table S1. Hierarchically nested general linear modelling (vegetation type nested in plot) detected significant differences of the Weibull fitting parameter (dependent variable) across all plots but not of vegetation type within plots. Species richness served as co-variate. Given are partial η^2 values and the coefficient of determination r^2 of the model. Parametric significances: ***: P < 0.001. N = 1725.

Variable	Nested in	df	partial η^2
Data set	-	47	0.23***
Vegetation type	data set	85	0.06
Species richness	-	1	<0.01
r^2	-	1591	0.49***

Table S2: Proportions of correctly identified group memberships for as Weibull parameter (φ, λ) – ln cover / species richness combinations shown in Figs. 2 (single plots) and 3 (cumulative plot sequences CPS). Groups were defined on the λ - ln(cover) relationships (Figs. 2a, 3a)

	Group	φ - ln (cover)	λ - ln(cover)	φ - species	λ - species
Plots	A	0.55	1.00	0.38	0.52
	B	0.55	0.96	0.47	0.60
	C	0.58	1.00	0.41	0.65
	D	0.52	0.93	0.59	0.60
CPS	A	0.47	1.00	0.45	0.51
	B	0.62	0.89	0.45	0.48
	C	0.61	0.97	0.47	0.53
	D	0.96	1.00	0.85	1.00

Table S3. Discriminant analysis (N = 442 plots for which all soil data were available) of the four SAD groups pointed to soil depth and organic matter content (OMC) as being most important determinants of SAD group membership in Palaearctic grasslands. T_{mean} and P_{mean} refer to mean annual temperatures and precipitation, respectively. The dominant eigenvector (EV1) of the geographic distance matrix, species richness, and ln-transformed total cover served as co-variates. Parametric significances: **: P < 0.01, ***: P < 0.001

Variable	Wilk's λ	Tolerance
T_{mean}	0.65	0.62
P_{mean}	0.65	0.72
Soil depth	0.67***	0.81
OMC	0.73***	0.72
EV1	0.70***	0.55
Species richness	0.66**	0.77
ln C	0.65	0.76

Table S4. Hierarchically nested general linear modelling (vegetation type and species ln-transformed cover *C* nested in plot) did not detect significant non-linear dependencies of empirical SAD skewness (γ) and kurtosis (δ) and Weibull shape (φ) and scale (λ) parameters with increasing C across all plots. Given are partial η^2-values and the coefficient of determination r^2 of the model. Parametric significances: *: P < 0.05, **: P < 0.01, ***: P < 0.001. N = 1719.

Variable	Nested in	df	γ	δ	φ	λ
Data set		45	0.34***	0.11***	0.30***	0.47***
Vegetation type	Data set	83	0.22	0.05	0.06	0.08**
C	Vegetation type	19	0.02*	0.01	0.03**	0.04***
C^2	Vegetation type	18	0.01	0.01	0.01	0.01
r^2			0.48***	0.18***	0.47***	0.68***

Table S5. Breakpoint analysis of 40 CPSs with at least 15 individual plots. Given are plot name and minimum (Y_{min}), breakpoint (Y_B), maximum (Y_{max}), and the X-value of the breakpoint (B) values of SAD skewness (γ), kurtosis (δ), and Weibull shape (φ), and scale parameters (λ) for the parameter – ln-abundance piecewise linear regressions. Shape (**I**ncrease, **D**ecrease, **U**-shaped, **I**nverse **U**-shaped) refers to the pattern of the segmented parameter – ln-abundance regression. Bold type indicates significant breakpoints at $P < 0.001$.

Plot	Grassland type	N	γ					δ					φ					λ				
			Y_{min}	Y_B	Y_{max}	B	Shape	Y_{min}	Y_B	Y_{max}	B	Shape	Y_{min}	Y_B	Y_{max}	B	Shape	Y_{min}	Y_B	Y_{max}	B	Shape
AT_E	A.4 Rocky	22	0.28	-0.27	-0.21	**6.62**	U	2.05	2.01	3.01	**5.29**	U	1.85	2.35	3.67	**5.75**	I	3.59	4.69	6.54	5.93	I
AT_E	B.2 Meso-xeric	18	0.21	-0.38	-0.15	**6.43**	U	1.70	2.72	2.53	**6.70**	IU	1.81	3.34	2.78	**7.26**	IU	3.27	6.36	4.33	**7.68**	IU
BG_A	A.4 Rocky	29	0.37	-0.33	-0.18	**5.30**	U	1.80	2.35	3.15	5.23	I	1.49	2.89	4.07	6.00	I	2.25	3.19	6.35	4.70	I
BG_A	B.2 Meso-xeric	20	0.49	0.36	0.18	6.34	D	4.04	2.48	2.62	**6.75**	U	2.17	2.48	2.76	**5.67**	I	3.98	4.88	5.27	**6.98**	I
BG_A	B.5 Mediterranean	17	0.21	0.18	0.43	**5.81**	U	2.12	1.78	2.28	**5.54**	U	1.94	2.11	1.90	**5.96**	IU	2.73	3.43	3.56	**6.34**	I
CH_C	B.3 Mesic	27	-0.83	-0.30	-1.10	5.64	IU	3.28	3.16	4.53	5.83	U	3.12	2.96	4.93	**6.51**	U	4.82	3.96	6.59	**6.36**	U
CH_D	A.4 Rocky	47	-0.57	-0.94	-0.11	4.55	U	2.34	3.50	2.91	5.03	IU	2.66	3.85	3.32	**5.84**	IU	2.26	5.30	6.79	**5.46**	I
CH_D	A.3 Xeric	40	-0.60	-0.44	-0.49	5.78	IU	2.71	3.35	3.03	**7.32**	IU	2.60	3.35	3.23	**5.97**	IU	4.28	5.83	6.81	**6.40**	I
CH_D	B.2 Meso-xeric	33	-0.08	-0.76	-0.59	**5.86**	U	4.06	4.26	3.49	**7.00**	IU	2.82	4.16	3.46	**6.59**	IU	3.71	5.26	6.85	**5.71**	I
DE_C	C.3_Saline	44	-0.12	-0.50	-0.21	**8.46**	U	2.15	2.50	2.26	**8.48**	IU	2.78	3.14	3.38	**8.33**	I	5.89	5.86	6.00	**8.47**	U
DE_F	B.3 Mesic	32	-0.44	-0.17	-0.77	5.58	IU	2.23	2.45	3.19	**7.54**	I	2.36	3.64	3.41	**8.03**	IU	3.32	6.10	5.80	**8.09**	IU
ES_A	B.2 Meso-xeric	28	-0.06	-0.48	-0.25	5.81	U	2.36	2.78	2.64	5.76	IU	2.08	3.47	3.41	**6.68**	IU	3.58	5.55	6.81	5.88	I
ES_A	A.4 Rocky	19	-0.16	-0.03	-0.24	4.76	IU	1.89	2.73	2.63	**7.18**	IU	2.21	2.74	2.95	**6.12**	I	5.01	5.73	6.31	6.20	I
EU_M	C.5 Wetlands	33	0.37	0.51	0.38	**7.39**	IU	2.77	1.99	2.38	4.85	U	1.90	1.87	2.10	**5.84**	U	2.62	2.48	4.10	4.93	U
HU_I	B.2 Meso-xeric	23	1.09	1.03	0.50	**6.63**	D	3.19	3.28	2.95	**6.67**	IU	1.26	1.33	1.75	**6.27**	I	1.94	2.27	3.40	**6.27**	I
IT_A	B.5 Mediterranean	22	0.19	-0.43	-0.09	**4.77**	U	2.60	2.73	2.57	7.52	IU	2.26	2.92	2.80	**5.36**	IU	3.08	3.65	4.48	**5.58**	I
IT_L	A.3 Xeric	32	-0.48	-0.44	-0.27	5.86	I	2.51	2.12	2.36	**7.12**	U	2.45	2.63	2.64	8.29	I	4.79	5.91	6.28	**7.02**	I
IT_L	B.2 Meso-xeric	34	-0.14	-0.28	-0.27	7.63	U	2.10	2.66	2.47	**7.53**	IU	2.55	3.01	2.69	**7.17**	IU	5.51	6.05	6.54	**6.76**	I
IT_T	B.3 Mesic	17	0.52	0.11	0.02	5.70	D	2.56	2.68	2.24	**6.48**	IU	1.82	3.24	2.98	**7.28**	IU	2.84	6.11	6.41	**8.84**	I
PL_A	B.2 Meso-xeric	48	0.28	-0.34	-0.42	**6.72**	D	3.11	2.66	2.83	**7.17**	U	2.58	2.63	2.70	5.96	I	4.07	5.25	5.75	**8.09**	I
PL_C_2012	B.3 Mesic	42	-0.15	-0.08	-0.29	**7.47**	IU	1.66	2.03	2.00	**6.83**	IU	2.00	2.45	2.50	**8.20**	I	5.57	5.07	6.91	**6.72**	U
PL_C_2013	B.3 Mesic	73	0.77	-0.25	-0.18	**7.49**	U	1.90	1.56	2.15	**6.73**	U	1.15	2.39	2.40	**7.93**	I	2.46	3.90	4.82	**7.35**	I
PL_C_2014	B.3 Mesic	101	1.03	0.27	0.09	**7.00**	D	2.15	2.21	2.26	-0.11	I	0.76	3.10	3.36	**7.27**	I	1.08	6.27	7.46	**7.19**	I

PL_C_2014.	B.4_Wet	17	0.39	0.54	0.11	6.03	IU	2.50	1.77	1.64	**6.67**	D	2.81	2.23	2.65	**6.30**	U	5.48	4.44	6.27	6.29	U
RO_C	B.2 Meso-xeric	49	0.45	0.37	0.00	5.71	D	2.49	2.32	2.26	**7.80**	D	1.78	2.00	2.12	**8.63**	I	2.76	3.72	4.13	**8.63**	I
RO_C	A.3_Xeric	17	0.00	0.05	-0.11	**7.04**	IU	1.81	2.27	1.99	5.24	IU	1.89	1.95	1.73	7.87	IU	2.76	2.88	3.35	6.24	I
RS_A	A.4 Rocky	51	-0.43	-0.08	-0.10	**6.31**	IU	2.46	2.58	2.53	**6.10**	IU	2.88	2.78	3.36	5.01	U	3.61	4.77	6.89	5.84	I
RS_A	A.3 Xeric	37	0.21	-0.34	-0.13	**7.07**	U	1.98	2.28	2.60	**7.52**	I	2.33	3.09	3.24	**7.13**	I	5.20	6.12	6.62	**7.10**	I
RU_A	A.3 Xeric	81	0.18	-0.19	-0.32	6.61	D	2.08	2.31	2.95	**7.06**	I	1.66	2.78	4.18	6.75	I	3.28	4.86	7.38	6.61	I
RU_A	B.2 Meso-xeric	20	-0.31	-0.07	-0.30	6.00	IU	2.53	2.75	2.60	8.29	IU	2.18	3.41	2.88	**7.58**	IU	3.95	5.41	5.31	**7.48**	IU
TJ_A	B.3 Mesic	18	0.21	0.06	0.32	**6.69**	U	1.87	2.63	2.76	**6.85**	I	1.35	2.00	1.92	**6.93**	IU	2.04	2.31	3.34	5.41	I
TR_B	B.5 Mediterranean	99	-0.53	-0.13	0.05	**7.76**	I	3.49	2.35	2.48	**8.94**	U	3.36	3.40	3.23	**7.67**	IU	3.13	4.25	4.60	**8.90**	I
UA_AN	A.3 Xeric	25	0.70	0.15	0.29	**5.63**	U	2.12	2.67	2.64	**6.78**	IU	1.49	2.54	2.56	**6.70**	I	2.37	4.37	5.14	**6.32**	I
UA_AP	B.2 Meso-xeric	25	-0.18	0.28	0.20	**6.02**	IU	2.04	1.66	2.54	**5.83**	U	2.02	1.40	3.00	**5.79**	U	3.22	2.71	5.40	**6.06**	U
UA_AP	A.3 Xeric	75	-0.41	-0.13	0.02	**4.60**	I	3.05	2.45	2.72	**4.18**	U	2.34	2.72	2.77	**6.53**	I	3.57	4.18	5.18	**5.87**	I
UA_F	B.2 Meso-xeric	20	1.59	0.53	0.42	**4.98**	D	3.91	2.33	2.03	4.97	D	0.66	2.00	1.56	4.47	IU	0.35	0.89	1.69	**5.73**	I
UA_F	A.3 Xeric	34	1.58	0.49	0.12	**5.42**	D	3.83	2.12	2.04	**5.89**	IU	1.40	1.56	1.77	5.23	I	0.74	1.00	1.90	5.54	I
UA_F	A.4 Rocky	105	1.29	0.43	0.10	**4.83**	D	3.41	2.20	1.97	3.89	D	1.35	1.67	1.91	**7.75**	I	0.56	1.83	2.45	**7.68**	I
UA_G	A.3_Xeric	16	0.98	0.20	0.20	**4.89**	D	4.03	2.84	2.19	**4.78**	D	1.84	2.30	2.29	**5.57**	IU	2.88	3.82	4.80	5.15	I
UA_J	A.3 Xeric	60	-0.29	-0.10	0.03	5.41	I	2.11	3.06	2.77	**7.32**	IU	2.52	2.23	3.96	5.60	U	4.09	4.29	7.03	5.71	I

Figure S1. Map of study sites with site names (same as in Appendix S2: Table S1). Colours relate to the four groups of SAD form outlined in the results:

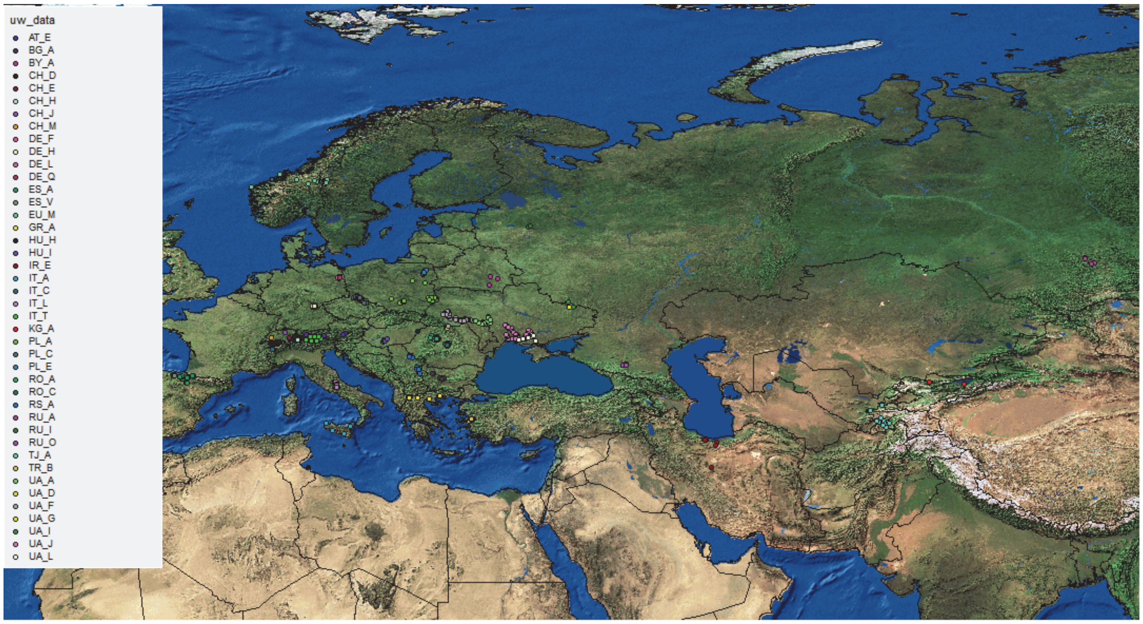

Figure S2. The quotient of Weibull shape and scale parameters (φ/λ) decreases with empirical SAD variances (σ^2) to a power function. Data from 1725 grassland plant communities. Red line: ordinary least squares power function fit to data, green line: respective fit to the data points at the lower boundary variances. The coefficient of determination refers to the red regression line.

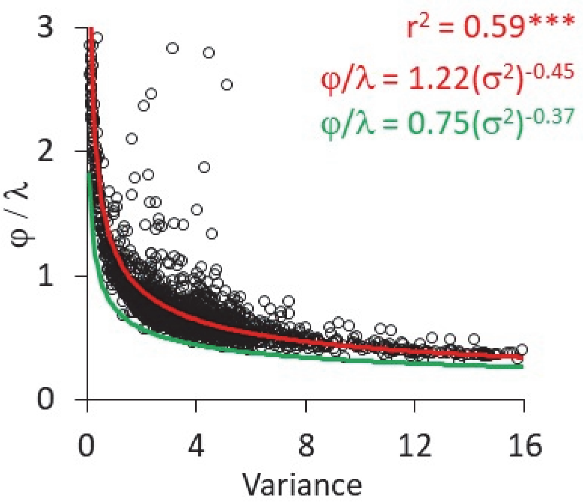

Figure S3. a: Numbers of plots per SAD group (red: group A, blue: B, green: C, yellow D) with respect to the 19 vegetation types. b, c: Median (with lower and upper 75 percentiles) of the goodness of fit measure of the four SAD groups (B) and vegetation types (C, only types represented by more than 15 communities). Blue, violet, and red vertical lines, respectively, denote the goodness of fit values 0.1 (excellent fit, Fig.1a of main text), 0.3 (very good, Fig. 1b, c), 0.5 (acceptable, Fig. 1d).

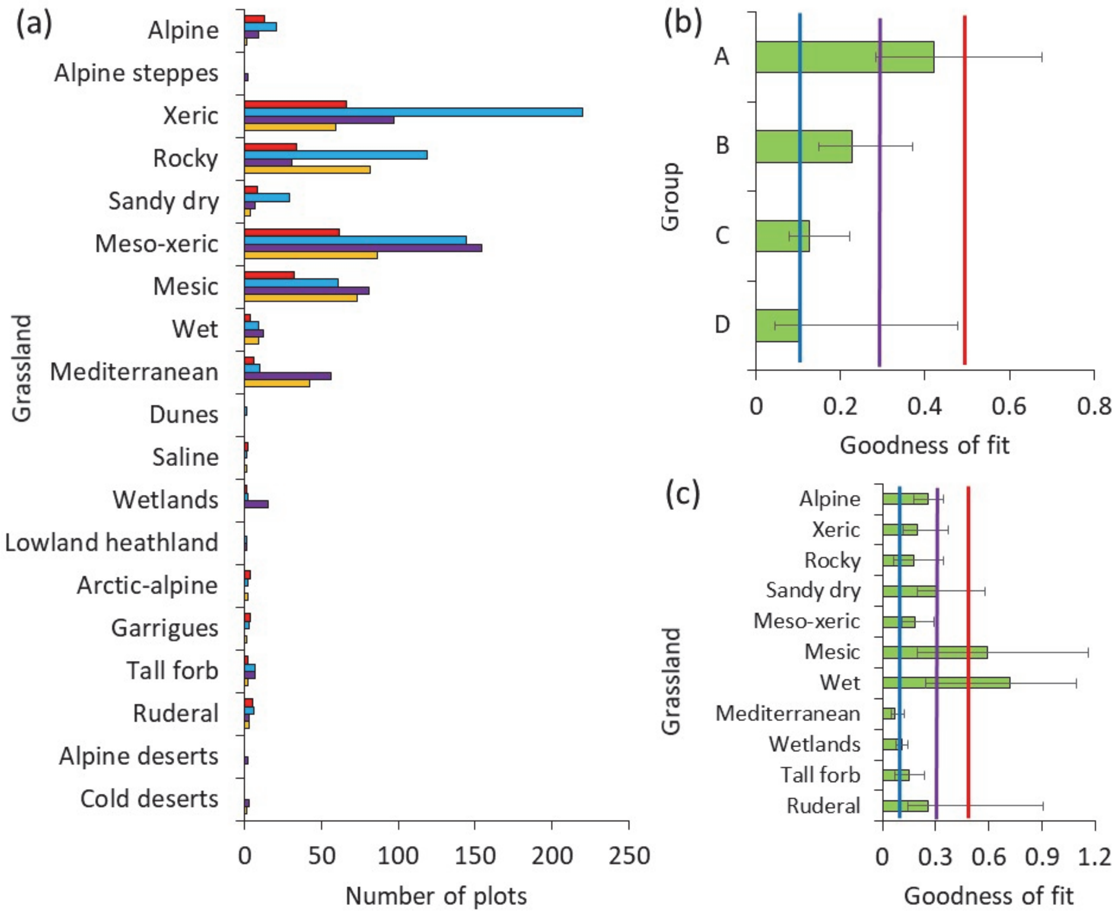

Figure S4. Empirical mean μ, variance σ^2, skewness γ and kurtosis δ of the ln-transformed relative abundance (cover values) distributions of 1725 grassland plant communities in dependence on total plant cover values (a, b) and species richness (c, d). Regression lines refer to logarithmic (a-d) and linear (e-h) OLS regressions. Parametric significances: ***: P < 0.01.

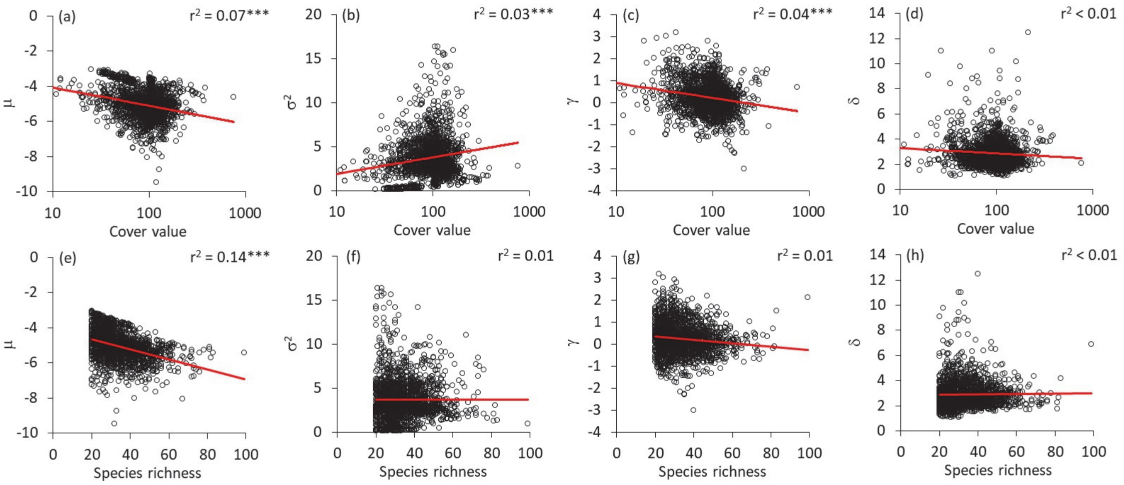

Figure S5. Plots of skewness (γ), kurtosis (δ) and of Weibull shape (φ) and scale (λ) parameters against basic geographical and climate characteristics (temperature in °C and annual precipitation in mm) of the study sites. r^2 values refer to ordinary linear least squares regressions.

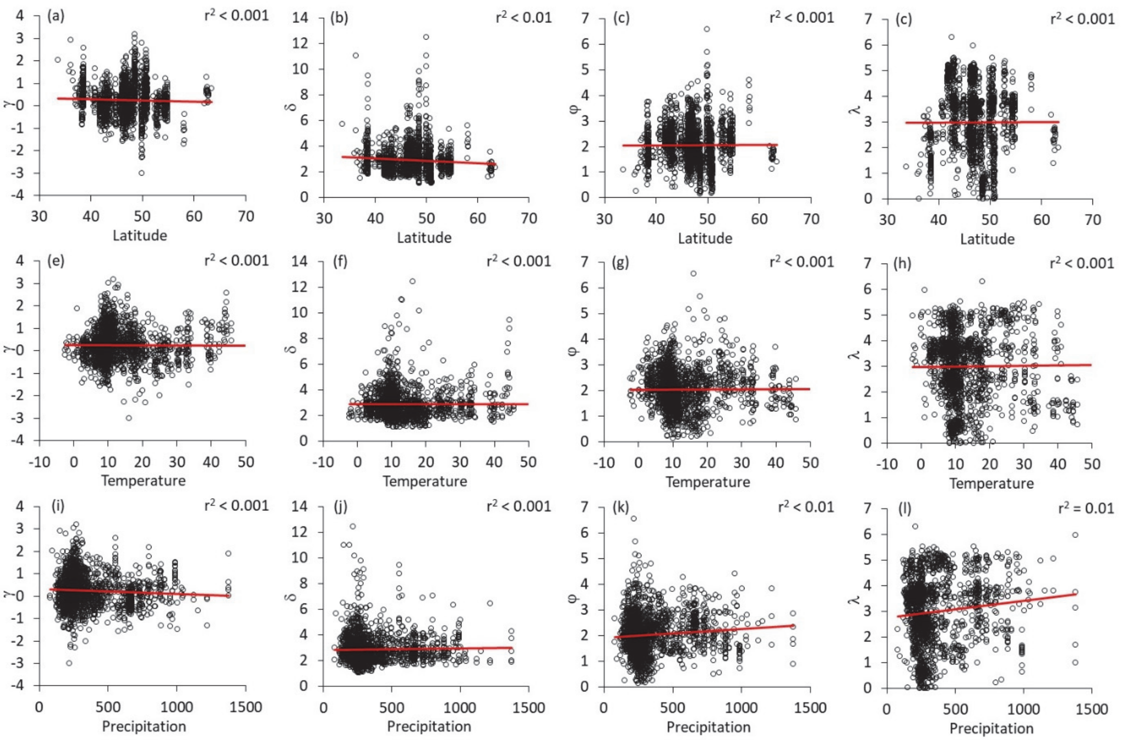

Figure S6. Plots of skewness (γ), kurtosis (δ) and of Weibull shape (φ) and scale (λ) parameters against soil depth in (cm), percentage organic matter content and the C/N ratio of the study sites. r^2 values refer to ordinary linear least squares regressions. Parametric significances: **: P < 0.01, ***: P < 0.001.

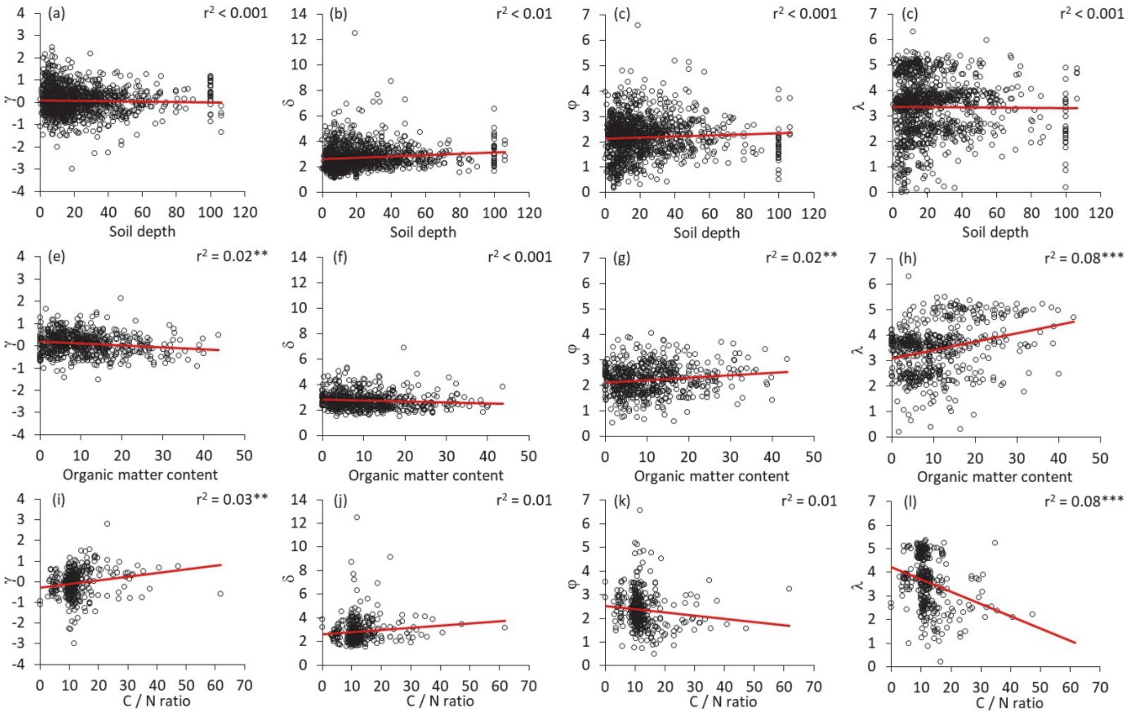

Supplement: Supplementary file 1 — Appendix S1 [file ECY-103-e3725-s001.pdf]
